# Elbow Extension Predicts Motor Impairment and Performance after Stroke

**DOI:** 10.1155/2011/381978

**Published:** 2011-02-06

**Authors:** Crystal L. Massie, Stacy Fritz, Matthew P. Malcolm

**Affiliations:** ^1^Health and Exercise Science Department and Occupational Therapy Department, Colorado State University, Fort Collins, CO 80523, USA; ^2^Physical Therapy Program, Department of Exercise Science, University of South Carolina, Columbia, SC 29208, USA; ^3^NeuroRehabilitation Research Laboratory, Occupational Therapy Department, Colorado State University, 219 Occupational Therapy Building, Fort Collins, CO 80523, USA

## Abstract

*Background and Purpose*. Kinematic motion analysis has helped to characterize poststroke reaching strategies with the hemiparetic arm. However, the relationships between reaching strategy and performance on common functional outcome measures remain unclear. *Methods*. Thirty-five participants were tested for motor performance and motor impairment using the Wolf Motor Function Test (time and functional ability measure) and Fugl-Meyer assessment, respectively. Kinematic motion analysis of a forward reaching paradigm provided potential predictors of reaching strategy including shoulder flexion, elbow extension, and trunk displacement. A stepwise linear regression model with three potential predictors was used in addition to Pearson-product moment correlations. *Results*. Kinematic analysis of elbow extension predicted performance on both the Wolf Motor Function Test and Fugl-Meyer assessment. Shoulder flexion and trunk displacement did not significantly predict functional or reaching time outcomes. The Wolf Motor Function Test and the Fugl-Meyer assessment were highly correlated. *Conclusions*. The ability to incorporate elbow extension during reach is a significant predictor of motor performance and hemiparetic arm motor capacity after stroke.

## 1. Introduction

Stroke is a common disabling condition that often impairs the ability to reach with the stroke-affected upper extremity. Because reaching is a necessary component of many tasks of daily living, survivors experience decreased autonomy and quality of life [1]. Recent conceptual shifts in stroke rehabilitation have stimulated an increase in upper-extremity interventions that are based on motor learning, motor control, and recovery of movement owing to activity-dependent neuroplasticity. More emphasis on the outcome measures used to establish improvements after intervention has also occurred in an effort to delineate motor recovery and/or compensation [2]. 

Many stroke rehabilitation outcomes have limited objective ability to characterize movement strategies [2, 3]. Outcome measures used in intervention research are often focused on task completion or clinician ratings of movement, resulting in limited, specific, precise, and quantitative data that effectively distinguishes remediation of deficits versus the development of compensatory movement strategies. The functional significance of a stroke survivor's ability to complete meaningful tasks should not be undermined, yet these types of outcome measures do not provide information regarding specific movement strategies [3]. The Wolf Motor Function Test (WMFT) is a common task-based outcome measure that has quickly become one standard measure in research investigations of upper-extremity rehabilitation interventions such as constraint-induced therapy (CIT). The WMFT incorporates gross- and fine-motor components of all joints in a variety of functional tasks such as reaching for a can, picking up a pencil, or folding a towel. The instructions for each task emphasize speed of completion and all tasks are videotaped for subsequent rating of functional ability. Functional ability is rated on a 6-point ordinal scale that incorporates task completion and generalizations regarding movement strategies (e.g., movements made in synergy). The WMFT also includes two strength measures but these are reported less in the scientific literature. The WMFT has established reliability [4–7]. The Fugl-Meyer Upper Extremity Assessment (FM) is another common measurement tool used in stroke rehabilitation. In addition to evaluating some basic movement tasks or task components (e.g., gripping a can or ball, holding a pencil with a two-point pinch), the FM assessment also evaluates more basic movement capacities foundational to task performance on a 3-point ordinal scale. For example, subjects are instructed to produce isolated shoulder movements while maintaining elbow extension during which an evaluator rates movement capacity. Other scored criteria include the presence of reflexes, tremor, dysmetria, and speed of movement.The FM has established validity and reliability as a research tool [8–10]. Together, the WMFT and FM assessments provide valuable information regarding motor performance and motor impairment after stroke, yet they do not yield precise quantitative data on movement strategies and may lack sufficient sensitivity to characterize changes in strategies over time. 

Levin et al. [2] suggested that more robust measures of movement strategy can be implemented in order to clarify recovery versus compensatory movement patterns after intervention. Kinematic motion analysis affords the ability to precisely quantify movement strategies during forward reach in survivors of stroke. Indeed, many studies have documented the presence of impaired reaching ability and inefficient compensatory movement after stroke [1, 11–13]. Motor control impairments include abnormal inter-joint coordination, decreased peak reaching velocity, and decreased movement smoothness. Forward reaching distance is also reduced following a stroke [14], presumably related to common flexor synergy patterns and a requirement to flex the shoulder against gravity [15]. The recruitment of anterior trunk flexion or increased shoulder abduction can then compensate for the limited elbow extension and shoulder flexion, respectively. These compensatory strategies may reflect learned responses to initial deficits that enable the attainment of a goal, yet may alter motor performance towards long-term inefficient and ineffective functional movements [11, 16]. 

As research to develop improved movement-related interventions rapidly grows, investigators and clinicians must also have precise and quantifiable evidence of how individuals with motor deficits accomplish movement. This is particularly important to ensure that rehabilitation strategies truly help the individual to achieve necessary efficiency, flexibility, and functional success when attempting to complete meaningful tasks. For example, while the WMFT yields performance time data for tasks involving hemiparetic reach, specific data on reaching strategies is not conveyed. As a result the relationship between movement strategy (e.g., the use of anterior trunk flexion or ability to extend the elbow) and task performance requiring forward reach is not clear. The purpose of this study was therefore to determine the relationship between reaching strategy and common task and motor capacity-based outcomes applied in stroke rehabilitation. Specifically, we used kinematic motion analysis to assess shoulder, elbow, and trunk contributions during hemiparetic reach, and the WMFT and FM to assess task performance and motor capacity as related to functional reach.

## 2. Methods

### 2.1. Participants

A convenience sample of 35 participants was used for this study. All participants gave written consent in accordance with the policies of the local institutional review board. Participants met the following inclusion criteria: at least 6 months after stroke; had at least 10° of active wrist extension and 10° of extension in 2 fingers and thumb; approximately 30° of active shoulder flexion; at least half the normalpassive range of motion at all upper-extremity joints. Exclusion criteria included other neurologic conditions (e.g., multiple sclerosis, Parkinson's disease); injections treating spasticity within 3 months of participation; a Mini-Mental State Exam score less than 24 [17]; a pain scored greater than 5 on the McGill Pain Scale. These criteria are similar to those applied in intensive upper limb stroke therapies, such as constraint-induced therapy [18]. 

### 2.2. Experimental Design

Participants underwent functional and kinematic motion analysis testing on the same day. Functional outcome measures included the Fugl-Meyer Assessment (FM), the Wolf Motor Function Test (WMFT) time, and functional ability scores. The time scores represent the average time to complete a task; if a subject was unable to complete the task, a maximum time of 120 seconds was used in the average. Three potential predictors of functional outcome measures were derived from kinematic motion analysis of hemiparetic reach: shoulder flexion, elbow extension, and anterior trunk displacement. 

### 2.3. Potential Predictors

**Figure 1:**
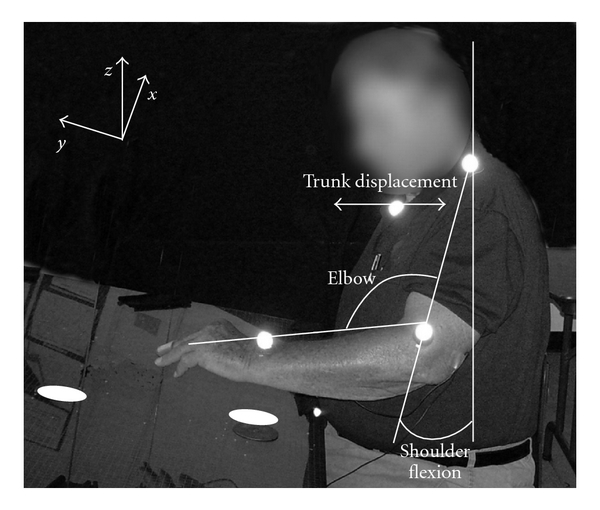
Experimental setup. Seated individuals reached with the stroke-affected arm between a proximal target and a distal target placed at maximum reach of the stroke-affected arm in a sagittal plane. Participants were instructed to tap back and forth as fast as they could, alternating between proximal and distal targets.

Three potential predictors were derived from kinematic motion analysis of the stroke-affected upper-extremity during reach: elbow flexion-extension, shoulder flexion-extension, and trunk displacement. Flexion-extension movements at the elbow and shoulder were quantified during a reaching task as these movements are core components of functional reach used during daily activities [1, 19]. Please see Figure 1 for experimental setup. Details of the kinematic reaching task have been reported elsewhere [20, 21]. The reaching task consisted of 4 flexion-extension movements alternating between the 2 targets positioned in the sagittal plane of the hemiparetic shoulder. The distal target was placed at the maximal reaching contact point, that is, the furthest point a subject could reach in the sagittal plane. The proximal target was placed at a natural returning position for the subject. Participants were instructed to reach between the two targets as fast as possible for a minimum of 4 reaching cycles. 

Arm kinematics were recorded at 60 Hz with a 3-dimensional camera-based motion analysis system. Reflective markers were placed on the sternal notch, shoulder, elbow, and wrist of the paretic arm. A sequence of 3-dimensional coordinates for each reflective marker, relative to the coordinate system built into the table surface,was calculated by the kinematic motion analysis software (Motus). Joint angles (shoulder flexion and elbow extension) were calculated as degrees of excursion by each joint when reaching from the proximal to the distal targets. Trunk anterior displacement was calculated as the linear distance that the sternal notch marker moved in the sagittal plane when the participant reached from the proximal to the distal target. Means for each segment were calculated based on the 4 reaching cycles.

### 2.4. Statistical Analysis

Descriptive statistics for the demographics, outcome measures, and potential predictors were calculated including the mean and standard errors for continuous data, and as counts for the categorical variables.

Normality of the outcome measures and potential predictors was statistically verified using the Shapiro-Wilks *W* test. The trunk displacement required transformation using the natural log to meet the assumptions of this test. The elbow extension excursions required transformation using the square root after a value of 4 was added to each value. The value of 4 was added to each score in order to make the data positive before the square root transformation was calculated. 

The Pearson-product moment correlation was calculated to determine correlations between the functional outcome measures and the kinematic measures. Potential predictor variables obtained with kinematic motion analysis were used to develop a general linear model for each of the dependent variables including FM scores and WMFT time and functional ability scores. A forward stepwise procedure was used in which each variable was examined at each step for entry into the model. Adjusted *R* ^2^ values and probability values were calculated. Presence of multicolinearity among predictor variables in the regression models was assessed using a variance inflation factor. Significance was set at *P* < .05.

## 3. Results

One participant was excluded from the study because her scores were within the 95% standard error of measure [7] of the normative data for her age group on the WMFT, indicating minimal or no impairment [22]. Descriptive statistics of the sample's demographics are listed in Table 1, and descriptive statistics for the dependent variables and the independent variables are listed in Table 2. 

**Figure 2:**
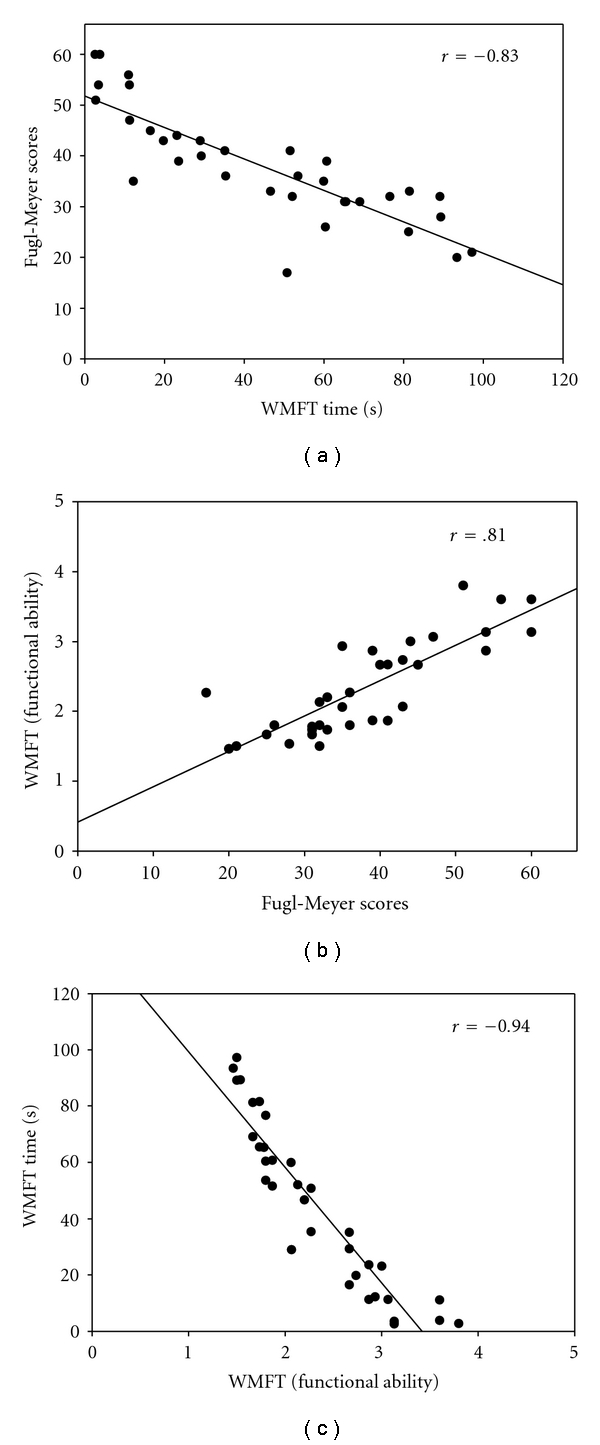
The correlations between the dependent measures (WMFT functional ability and time scores and the FM). The associations were all strongly associated and were significant at *P* < .01.

Graphical displays of correlations between dependent variables are displayed in Figures 2(a)–2(c). WMFT time and FM scores were strongly negatively correlated (*r* = − .83) indicating that faster performance time on the WMFT was associated with a higher FM total score. The correlation between the WMFT functional ability and FM scores was (*r* = .81) indicating that higher FM scores were associated with higher functional ability scores. The WMFT functional ability and time scores were strongly negatively correlated (*r* = −.94). 

**Figure 3:**
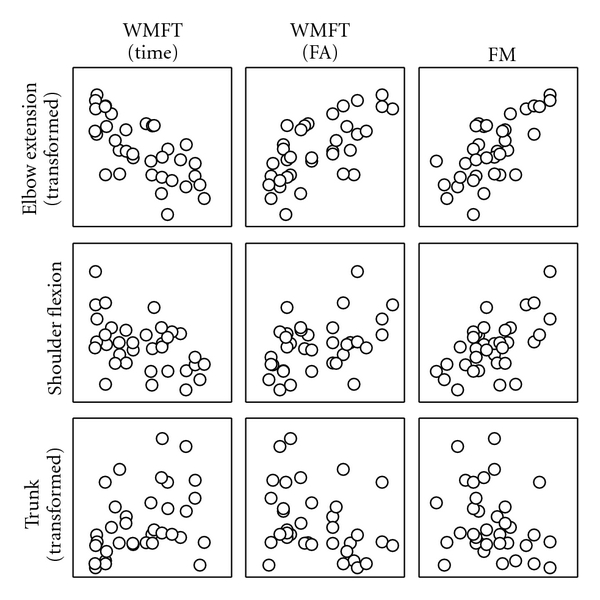
A scatterplot matrix of the kinematic predictor variables with the outcome measures.

As demonstrated in Figure 3 and Table 3, elbow extension was strongly correlated with WMFT time (*r* = −.69), WMFT functional ability (*r* = .67) and FM score (*r* = .70). The potential predictors were entered into three linear multiple regression models with stepwise entry using the WMFT time and functional ability scores and FM scores as the dependent variables. The only significant predictor for the WMFT time scores and the FM scores was the amount of elbow extension. Shoulder flexion and anterior trunk movement were removed during the regression analysis. The elbow accounted for 0.464 of the variance in the WMFT time scores, 0.46 of the variance of the WMFT functional ability scores, and 0.477 of the variance of the FM scores. The final regression equations are as follows:

(1) $$\begin{array}{c} \text{WMFT}\;{\left(\text{time}\right)}^{\prime }=87.2-9.3\;\left[\text{square}\;\text{root}\;\left(\text{elbow}+4\right)\right],\\ \text{WMFT}\;{\left(\text{fa}\right)}^{\prime }=1.4+0.217\;\left[\text{square}\;\text{root}\;\left(\text{elbow}+4\right)\right],\\ {\text{FM}}^{\prime }=18.3+3.1\;\left[\text{square}\;\text{root}\;\left(\text{elbow}+4\right)\right]. \end{array}$$

These equations could be used to model the predicted functional scores based on the amount of elbow extension when reaching between two targets as described in the kinematics task. Note that the amount of elbow extension entered into the equation requires the appropriate transformation (i.e., adding a value of 4 and taking the square root).

## 4. Discussion

Survivors of stroke often develop stereotypical movement patterns including a limited ability to extend the elbow and an increased reliance on anterior trunk flexion during forward reach. These are clinically understood to result from dominating motor synergies and weakness and commonly result in the survivor of stroke learning compensatory movements to try to accomplish functional and meaningful tasks. The inefficiency of compensatory movements represents one obvious contributor to decreased hemiparetic limb use during daily routines. Although intensive rehabilitation approaches such as CIT have successfully demonstrated success in ameliorating learned nonuse associated with upper limb hemiparesis, the intervention has been less focused on measuring changes in (and perhaps improving) specific movement strategies. Indeed, much weight has been given to outcome measures concerned with performance time and general upper limb motor capacity, without connecting these data to measures of specific movement strategies. As a result, studies may report subjects' performing faster, but lack a picture of how their movements “look”, that is, what movement strategies does the survivor of stroke adopt in order to decrease his or her performance time during reaching tasks? The findings from the current study indicate that faster task performance times and upper limb motor capacity are associated with subjects' ability to generate larger elbow extension excursions during hemiparetic reach. Furthermore, the amount of elbow extension used during forward reach may predict motor performance on the WMFT and motor impairment on the FM. Both sets of findings point to at least one specific movement strategy, that is, elbow extension, as related to and important for functional use of the hemiparetic arm. 

Difficulty extending the elbow after stroke is common and this limitation is clinically observed as part of a flexor synergy pattern that produces concurrent flexion motions, and which also often impairs the survivor of stroke's ability to control individual joints [23]. Zackowski et al. [23] characterized the difficulty in extending the elbow as part of a “joint individuation deficit”, andhypothesized that this deficit was more correlated with abnormal reaching performance than other potential predictors such as impaired sensation. The results from the current study parallel this finding-elbow extension was strongly correlated with the functional outcome variables that included a reaching component. 

We found that both anterior trunk displacement and shoulder flexion were not predictive of functional outcome measures. This is an interesting finding because of expected relationships among movement characteristics. For example, the reduction in elbow extension may be compensated by an increase in shoulder flexion or anterior trunk flexion. However, the results from the current study suggest that there is no strong relationship between the amount of trunk use and functional performance (see Table 3). This finding further supports the concept that the trunk may not be an obligatory movement patterns after stroke because there is no association with these functional outcomes. These results differ withother reports on hemiparetic reach. For example, Michaelsen et al. [1] found that elbow extension predicted approximately 80% of the variance in trunk movement during a forward reaching task with the hemiparetic arm. In addition, Beebe and Lang [24] found that only shoulder and middle finger active range of motion at 1 month after stroke significantly explained the variance on upper extremity function at 3 months after stroke. While we obviously discovered a different finding than these reports, methodological differences may explain the nonparallel findings between these studies and the present one. The present study limited the number of potential predictors to three to maintain an adequate sample size for each predictor. We also incorporated predictor variables based upon a dynamic reaching paradigm rather than isolating movement at each joint, as previous work has done. A dynamic model arguably approximates functional use of the hemiparetic arm better than one that only considers isolated movements at a particular joint. In other words, the contributions of shoulder flexion and trunk displacement were clearly less than elbow extension during reach when a dynamic reaching model was applied in our study. Additionally, differences in the characteristics of the subjects in the present study compared to these other studies may limit these interpretations given that the functional status may have differed. 

Difficulty extending the elbow is a clearly documented result of stroke, which intuitively impacts performance on functional outcome measures used in stroke rehabilitation interventions. Both the WMFT and FMare functional outcome measures that require a certain amount of elbow extension during components of each assessment. This is likely one reason that our finding that elbow extension significantly predicted performance on these two outcomes. Some tasks within the FM require isolated shoulder movements (flexion and abduction), and the ability to achieve and maintain elbow extension during those movements is a critical part of the scoring. Other tasks require the ability to maintain the correct elbow position while isolating movements at the wrist. The emphasis on the control of the elbow joint in the FM may explain why the ability to use elbow extension significantly predicted level of impairment. Many of the tasks on the WMFT require some degree of elbow extension during forward reach. For example, subjects are asked to reach from their lap to the table during all of the fine-motor tasks. Another task also requires elbow extension to slide the hemiparetic hand towards a lateral target line. The interesting finding is that elbow extension explained approximately the same degree of variability in the FM and WMFT scores, yet these two outcomes require quite different control of the elbow joint. The FM requires the elbow joint to be stabilized in extension, whereas the WMFT requires more active elbow flexion and extension during functional tasks. This suggests that the ability to control the elbow joint is critical for evaluation of motor capacity as well as motor task performance. 

This study is not without limitations. First, the number of potential predictors entered into the regression models were limited by the sample size (i.e., 10–20 subjects per predictor). For this reason, only trunk, shoulder, and elbow kinematic predictors were entered into the regression models. The potential exists for additional degrees of freedom to have explained more variance in the dependent variables. For example, the amount of wrist extension used may have influence performance. Second, data were only collected at baseline and do not allow for the predictions over time. This is a future area of research that should be expanded in order to further elucidate how changes in reaching strategy impact performance (i.e., do compensatory movements lead to long-term consequences?). Finally, subjects represent only a subset of the stroke population that have some return of voluntary control and motor function of the stroke affected limb according to the motor inclusion criteria. These movement characteristics would be common in approximately 20% of the stroke populations [25]. A larger sample size would increase external validity by allowing for more generalizations to be made from this research, that is, to a population presenting with varying degrees of motor impairment. 

## 5. Conclusion

In conclusion, the results of this study suggest that movement strategies must be more precisely examined and related to functional performance and capacity. Doing so would arguably assist rehabilitation scientists and clinicians in delineating motor recovery from compensatory patterns of movement and would hopefully influence the development and administration of therapeutic interventions. Further, in addition to CIT's emphasis on increasing amount of hemiparetic arm use, these data indicate that a participant's adopted movement strategies are measured in relation to functional performance and also identified as another agent of change in the rehabilitation process.

## Figures and Tables

**Table 1:** Demographics of participants (*n* = 34).

Age, years	$\overset{®}{X}=\;59.7\;(\pm \;16.3)$
Time since stroke, years	$\overset{®}{X}=3.0\;(\pm \;4.3)$
Side of Infarct	18 LCVA; 16 RCVA
Sex	16 Female; 18 Male

LCVA: left cerebrovascular accident; RCVA: right cerebrovascular accident.

**Table 2:** Descriptive statistics (*n* = 34).

Outcome variables	Mean (St Err)	Transformed
Fugl-Meyer scores	38.0 (1.9)	
WMFT time (seconds)	44.5 (5.1)	
WMFT functional ability	2.3 (0.1)	

Potential predictors		
Shoulder flexion (degrees)	41.9 (3.3)	
Elbow extension (degrees)	21.6 (3.2)	4.7 (0.3)
Trunk anterior displacement (cm)	6.8 (40.7)	1.7 (0.1)

**Table 3:** Pearson correlation coefficients of functional and kinematic measures.

Functional measure	Kinematic measures		
	Shoulder flexion	Elbow extension	Anterior trunk flexion
WMFT time (sec)	−.47**	−.69**	.37
WMFT (functional ability)	.42	.67**	−.34
Fugl-Meyer	.59**	.70**	−.29

**P < .01.

## References

[1] Michaelsen SM, Jacobs S, Roby-Brami A, Levin MF (2004). Compensation for distal impairments of grasping in adults with hemiparesis. *Experimental Brain Research*.

[2] Levin MF, Kleim JA, Wolf SL (2009). What do motor "recovery" and "compensationg" mean in patients following stroke?. *Neurorehabilitation and Neural Repair*.

[3] Wagner JM, Lang CE, Sahrmann SA, Edwards DF, Dromerick AW (2007). Sensorimotor impairments and reaching performance in subjects with poststroke hemiparesis during the first few months of recovery. *Physical Therapy*.

[4] Taub E, Uswatte G, Pidikiti R (1999). Constraint-induced movement therapy: a new family of techniques with broad application to physical rehabilitation—a clinical review. *Journal of Rehabilitation Research and Development*.

[5] Kunkel A, Kopp B, Müller G (1999). Constraint-induced movement therapy for motor recovery in chronic stroke patients. *Archives of Physical Medicine and Rehabilitation*.

[6] Morris DM, Uswatte G, Crago JE, Cook EW, Taub E (2001). The reliability of the wolf motor function test for assessing upper extremity function after stroke. *Archives of Physical Medicine and Rehabilitation*.

[7] Fritz SL, Blanton S, Uswatte G, Taub E, Wolf SL (2009). Minimal detectable change scores for the wolf motor function test. *Neurorehabilitation and Neural Repair*.

[8] Duncan PW, Propst M, Nelson SG (1983). Reliability of the Fugl-Meyer assessment of sensorimotor recovery following cerebrovascular accident. *Physical Therapy*.

[9] Platz T, Pinkowski C, van Wijck F, Kim INH, di Bella P, Johnson G (2005). Reliability and validity of arm function assessment with standardized guidelines for the Fugl-Meyer Test, Action Research Arm Test and Box and Block Test: a multicentre study. *Clinical Rehabilitation*.

[10] Sanford J, Moreland J, Swanson LR, Stratford PW, Gowland C (1993). Reliability of the Fugl-Meyer assessment for testing motor performance in patients following stroke. *Physical Therapy*.

[11] Levin MF, Michaelsen SM, Cirstea CM, Roby-Brami A (2002). Use of the trunk for reaching targets placed within and beyond the reach in adult hemiparesis. *Experimental Brain Research*.

[12] Levin MF, Selles RW, Verheul MHG, Meijer OG (2000). Deficits in the coordination of agonist and antagonist muscles in stroke patients: implications for normal motor control. *Brain Research*.

[13] Roby-Brami A, Feydy A, Combeaud M, Biryukova EV, Bussel B, Levin MF (2003). Motor compensation and recovery for reaching in stroke patients. *Acta Neurologica Scandinavica*.

[14] Kamper DG, McKenna-Cole AN, Kahn LE, Reinkensmeyer DJ (2002). Alterations in reaching after stroke and their relation to movement direction and impairment severity. *Archives of Physical Medicine and Rehabilitation*.

[15] Ellis MD, Sukal T, DeMott T, Dewald JPA (2008). Augmenting clinical evaluation of hemiparetic arm movement with a laboratory-based quantitative measurement of kinematics as a function of limb loading. *Neurorehabilitation and Neural Repair*.

[16] Woodbury ML, Howland DR, McGuirk TE (2009). Effects of trunk restraint combined with intensive task practice on poststroke upper extremity reach and function: a pilot study. *Neurorehabilitation and Neural Repair*.

[17] Folstein MF, Folstein SE, McHugh PR (1975). ’Mini mental state’. A practical method for grading the cognitive state of patients for the clinician. *Journal of Psychiatric Research*.

[18] Wolf SL, Winstein CJ, Miller JP (2006). Effect of constraint-induced movement therapy on upper extremity function 3 to 9 months after stroke: the EXCITE randomized clinical trial. *Journal of the American Medical Association*.

[19] Thaut MH, Kenyon GP, Hurt CP, McIntosh GC, Hoemberg V (2002). Kinematic optimization of spatiotemporal patterns in paretic arm training with stroke patients. *Neuropsychologia*.

[20] Malcolm MP, Massie C, Thaut M (2009). Rhythmic auditory-motor entrainment improves hemiparetic arm kinematics during reaching movements: a pilot study. *Topics in Stroke Rehabilitation*.

[21] Massie C, Malcolm MP, Greene D, Thaut M (2009). The effects of constraint-induced therapy on kinematic outcomes and compensatory movement patterns: an exploratory study. *Archives of Physical Medicine and Rehabilitation*.

[22] Wolf SL, McJunkin JP, Swanson ML, Weiss PS (2006). Pilot normative database for the wolf motor function test. *Archives of Physical Medicine and Rehabilitation*.

[23] Zackowski KM, Dromerick AW, Sahrmann SA, Thach WT, Bastian AJ (2004). How do strength, sensation, spasticity and joint individuation relate to the reaching deficits of people with chronic hemiparesis?. *Brain*.

[24] Beebe JA, Lang CE (2009). Active range of motion predicts upper extremity function 3 months after stroke. *Stroke*.

[25] Hakkennes S, Keating JL (2005). Constraint-induced movement therapy following stroke: a systematic review of randomised controlled trials. *Australian Journal of Physiotherapy*.

